# Ferroptosis and circular RNAs: new horizons in cancer therapy

**DOI:** 10.17179/excli2024-7005

**Published:** 2024-04-25

**Authors:** Asif Ahmad Bhat, Neelima Kukreti, Muhammad Afzal, Ahsas Goyal, Riya Thapa, Haider Ali, Moyad Shahwan, Waleed Hassan Almalki, Imran Kazmi, Sami I. Alzarea, Sachin Kumar Singh, Kamal Dua, Gaurav Gupta

**Affiliations:** 1 School of Pharmacy, Suresh Gyan Vihar University, Jagatpura, Mahal Road, Jaipur, India; 2 School of Pharmacy, Graphic Era Hill University, Dehradun 248007, India; 3 Department of Pharmaceutical Sciences, Pharmacy Program, Batterjee Medical College, P.O. Box 6231, Jeddah 21442, Saudi Arabia; 4 Institute of Pharmaceutical Research, GLA University, Mathura, U. P., India; 5 Center for Global Health Research, Saveetha Medical College, Saveetha Institute of Medical and Technical Sciences, Saveetha University, India; 6 Department of Pharmacology, Kyrgyz State Medical College, Bishkek, Kyrgyzstan; 7 Department of Clinical Sciences, College of Pharmacy and Health Sciences, Ajman University, Ajman, 346, United Arab Emirates; 8 Centre of Medical and Bio-allied Health Sciences Research, Ajman University, Ajman, Ajman, 346, United Arab Emirates; 9 Department of Pharmacology, College of Pharmacy, Umm Al-Qura University, Makkah, Saudi Arabia; 10 Department of Biochemistry, Faculty of Science, King Abdulaziz University, 21589, Jeddah, Saudi Arabia; 11 Department of Pharmacology, College of Pharmacy, Jouf University, 72341, Sakaka, Al-Jouf, Saudi Arabia; 12 School of Pharmaceutical Sciences, Lovely Professional University, Phagwara 144411, India; 13 Faculty of Health, Australian Research Center in Complementary and Integrative Medicine, University of Technology, Sydney, Ultimo-NSW 2007, Australia; 14 School of Medical and Life Sciences, Sunway University, Sunway, Malaysia; 15 Discipline of Pharmacy, Graduate School of Health, University of Technology, Sydney, Ultimo-NSW 2007, Australia; 16 Uttaranchal Institute of Pharmaceutical Sciences, Uttaranchal University, Dehradun, India

**Keywords:** circRNAs, ferroptosis, cancer treatment, molecular regulation

## Abstract

Cancer poses intricate challenges to treatment due to its complexity and diversity. Ferroptosis and circular RNAs (circRNAs) are emerging as innovative therapeutic avenues amid the evolving landscape of cancer therapy. Extensive investigations into circRNAs reveal their diverse roles, ranging from molecular regulators to pivotal influencers of ferroptosis in cancer cell lines. The results underscore the significance of circRNAs in modulating molecular pathways that impact crucial aspects of cancer development, including cell survival, proliferation, and metastasis. A detailed analysis delineates these pathways, shedding light on the molecular mechanisms through which circRNAs influence ferroptosis. Building upon recent experimental findings, the study evaluates the therapeutic potential of targeting circRNAs to induce ferroptosis. By identifying specific circRNAs associated with the etiology of cancer, this analysis paves the way for the development of targeted therapeutics that exploit vulnerabilities in cancer cells. This review consolidates the existing understanding of ferroptosis and circRNAs, emphasizing their role in cancer therapy and providing impetus for ongoing research in this dynamic field.

See also the graphical abstract(Fig. 1).

## Introduction

Cancer is fundamentally a broad range of disorders characterized by unchecked cell growth, tissue invasion, and a tendency toward metastasis (Chen et al., 2021[43]). Complex connections within signaling networks, genetic abnormalities, and disrupted cellular procedures are the molecular underpinnings of cancer (Hassannia et al., 2019[92]). Conventional treatment techniques, such as radiation and chemotherapy, sometimes encounter difficulties in targeting individual cancer cells, necessitating a shift to more focused and precise treatments (Koppula et al., 2021[108]). Once relegated to genetic uncertainty, the elucidation of ncRNAs has revealed previously unknown regions of cellular control and disease causation (Lei et al., 2022[115]). Analysis has focused on circRNAs in particular because of their varied functional functions in modulating expression of genes (Jiang et al., 2022[103]). Because of their covalently closed loop structure, which makes them resilient and resistant to degradation, circRNAs may play a key role in orchestrating the control of cellular activities (Ju et al., 2023[104]). At the same time, scientists have become interested in ferroptosis, as it has emerged as a new type of con-trolled cell death with special (Li et al., 2023[125]). Ferroptosis, in contrast to more conventional forms such as necrosis or apoptosis, is based on the iron-dependent build-up of lipid peroxides, which ultimately results in cellular death and harm to membranes (Liu et al., 2023[131]). Ferroptosis, which was first linked to neurological disorders, has become relevant in the field of cancer biology and presents a viable method for the targeted destruction of cancer cells (Liu et al., 2023[133]). An interesting frontier where ncRNA regulation converges with apoptosis is the intersection of ferroptosis and circRNAs. Recent research highlights the critical functions that certain circRNAs play in regulating ferroptotic pathways and influencing the fate of cancer cells (Balihodzic et al., 2022[12]; Gao et al., 2022[76]). These complex regulatory processes include sequestering proteins, directly interacting with miRNAs, and modifying important signaling pathways linked to ferroptosis (Wu et al., 2021[218]). An abundance of promising beneficial targets for the cure or prevention of cancer is made possible by the investigation of ferroptosis and circRNAs (Wang et al., 2021[204]). Researchers anticipate a future where precision therapy takes advantage of the distinct vulnerabilities present in cancer cells by comprehending the molecular conversations driving these activities (Krzyszczyk et al., 2018[109]). A major step towards more potent and less hazardous cancer treatments is being taken with the potential method of selectively inducing cell death in malignant cells by targeting circRNAs to affect ferroptosis (Tang et al., 2021[193]). The opportunities that arise from understanding the interaction between ferroptosis and CircRNAs are perfectly in line with the paradigm of precision (Yang et al., 2023[227]). Adapting treatment plans in accordance with the unique genetic and molecular characteristics of each tumor offers individualized and successful cancer treatment plans (Malone et al., 2020[144]; Yang et al., 2023[228]). This strategy gains more precision with the discovery of circRNAs as ferroptosis regulators, which has the potential to completely alter the rehabilitation landscape (Li et al., 2023[120]).

## Understanding Circular RNAs

The inherent covalently closed loop structure of circRNAs distinguishes them as a class of ncRNAs characterized by exceptional stability and resilience against degradation (Wang et al., 2017[209]). In a variety of cell types and tissues, including the hematopoietic compartment, they are strictly regulated and widely expressed (Goodell et al., 2015[82]). To differentiate them from linear RNAs, circ-RNAs have a circular in shape. Back-splicing creates this circular shape by joining an upstream splice acceptor and downstream splice donor to create a covalently closed loop (Lasda and Parker, 2014[112]). CircRNAs are more stable than linear RNAs because of their special structure, which prevents them from being broken down by exonucleases and leaves them free of free ends (Ge et al., 2023[79]; Yin et al., 2022[230]). CircRNAs play a variety of roles in controlling how genes are expressed. By capturing microRNAs and preventing their regulatory interactions with target mRNAs linked to host genes, they operate as microRNA sponges (Ayaz et al., 2023[9]). Furthermore, circRNAs interact with proteins that bind RNA, modifying the availability and functional dynamics of these proteins (Zhang et al., 2022[235]). Furthermore, circRNAs may precisely control the expression of genes due to their ability to bind with proteins or DNA. A strong correlation has been shown in recent studies between circRNAs and the development of cancer (Liu et al., 2023[132]; Wang et al., 2022[205]). CircRNAs have been linked to drug concern, chemoresistance, and cancer stem cells, among other features of the disease (Akter et al., 2022[3]). CircRNAs have the ability to affect the onset and course of cancer by altering gene expression and engaging in competition with microRNAs for binding (Bhat et al., 2023[24]). Consequently, they are coming to light as significant contributors to human illness, especially when it comes to cancer. CircRNAs are now being investigated as possible targets for cutting-edge treatment strategies meant to reduce chemoresistance and influence the growth of cancer.

## Ferroptosis in Cancer

**Figure 2:**
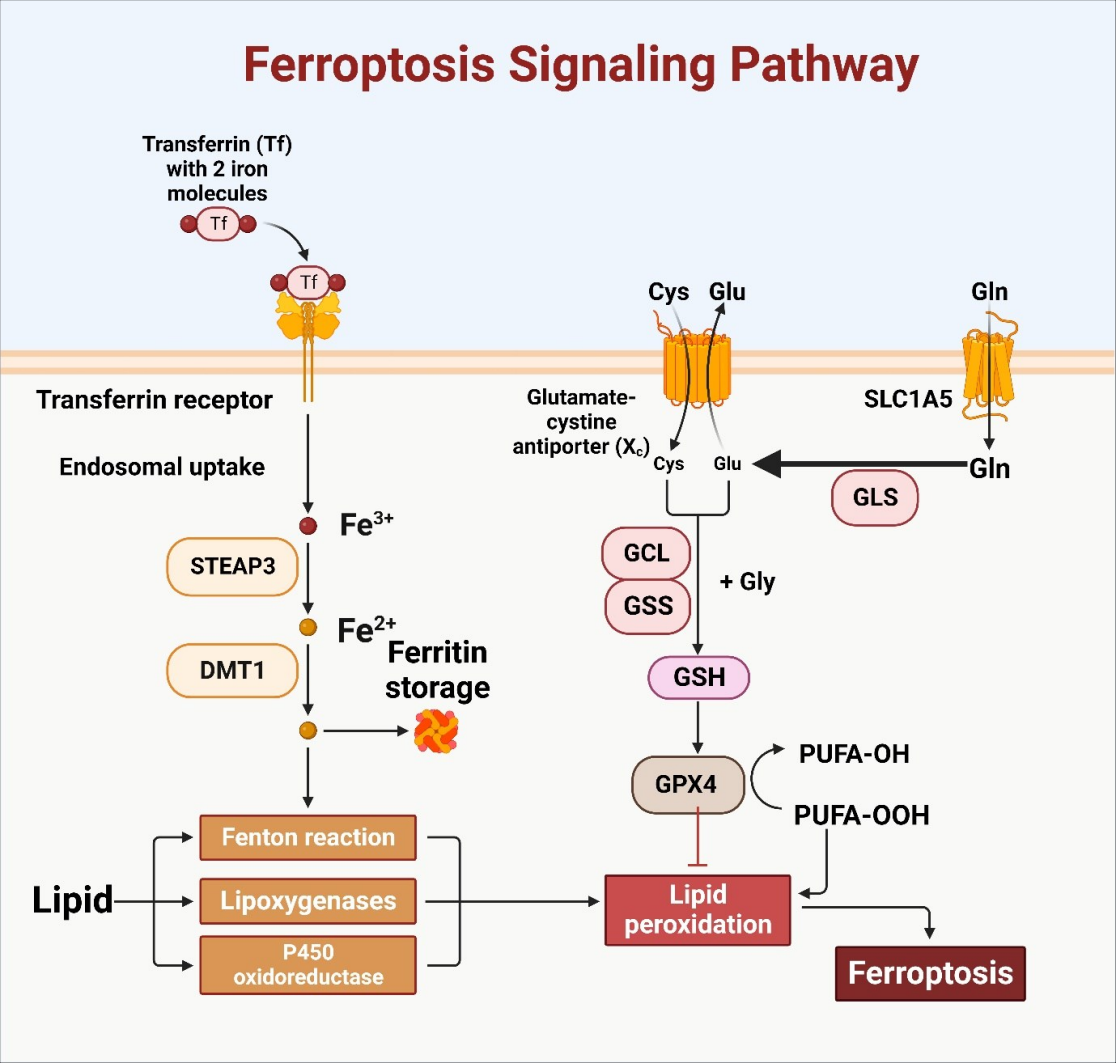
As picture depicted, through Six transmembrane epithelial antigen of the prostate (STEAP) and Divalent Metal Transporter 1 (DMT1), transferrin receptor-mediated iron absorption activates the ferroptosis signaling cascade. Intracellular iron levels rise as a result of this mechanism. Concurrently, the glutamine-cysteine antiporter facilitates cysteine import, speeding up the production of glutathione (GSH). GSH and glutathione peroxidase 4 (GPX4) work together to counteract lipid peroxidation, as illustrated. When this complex system is dysregulated, it can result in increased oxidative stress and ferroptosis, a critical phase in cancer.

Lipid peroxidation that is dependent on iron is a characteristic of ferroptosis. It has been connected to a number of cancer types and is important for cancer treatment (Zhang et al., 2022[236]). It is characterized by the iron-dependent build-up of lipid hydroperoxides, which causes cell death and harm to membranes (Yu et al., 2017[232]; Zhou et al., 2023[242]). The equilibrium between lipid peroxidation and repair mechanisms, exemplified by glutathione peroxidase 4 (GPX4) and the Xc-cysteine/glutamate antiporter system, plays a crucial role in controlling the entire process (Li et al., 2022[121]). Inducing ferroptosis emerges as a potentially effective cancer treatment method due to the heightened vulnerability of cancer cells, driven by their elevated metabolic demands and increased iron accumulation (Chen et al., 2023[46]). Additionally, specific cancer cells develop resistance to traditional treatments such as radiation and chemotherapy, while retaining their capability to trigger ferroptosis (Li et al., 2024[126]; Zhang et al., 2022[239]). Ferroptosis-inducing compounds are currently under investigation as potential anticancer treatments (Wu et al., 2020[219]). Studies have shown that ferroptosis is important for many kinds of cancer. For instance, the deregulation of ferroptosis-related pathways has been linked to treatment resistance and tumor growth in several forms of pancreatic, colon cancer, and pulmonary cancer (Yu et al., 2023[233]). Furthermore, in certain cancer types, the expression of important ferroptosis regulators such GPX4 and system Xc has been connected to therapy response and prognosis (Lu et al., 2017[136]; Zhu et al., 2021[243]). Developing therapy strategies and individualized treatment techniques requires an understanding of the unique functions that ferroptosis plays in various cancer situations (Luo et al., 2021[138]) (Figure 2(Fig. 2)).

## Intersection of circRNAs and Ferroptosis

### Gastric cancer

The cells lining the stomach are the source of gastric cancer, commonly referred to as stomach cancer (Douda et al., 2022[60]). The condition is intricate, featuring various subtypes, and its progression is influenced by factors such as genetics, lifestyle, nutrition, and the presence of *Helicobacter pylori* infection (Cover and Blaser, 2009[50]). Possible symptoms include indigestion, abdominal pain, unintentional weight loss, and blood in the stool, with the diagnosis involving imaging investigations, biopsies, and endoscopy (Narayanan et al., 2018[157]). Treatment include surgery, chemotherapy, radiation treatment, and targeted medications, depending on the stage of the malignancy (Debela et al., 2021[54]; Zhao et al., 2023[240]). Early diagnosis is crucial for improved outcomes, underscoring the importance of routine screenings and awareness of risk factors (Maxim et al., 2014[147]). With an expected 783,000 deaths in 2018, gastric cancer ranks as the third most fatal malignancy and the fifth most frequent neoplasm, posing a serious threat to world health (Rawla and Barsouk, 2019[171]). Each location has a different incidence and death rate, and nutrition and *Helicobacter pylori* infection play a major role (Franceschi et al., 2014[71]; Li et al., 2024[118]). Advances in the detection, management, and prevention of *H. pylori* have reduced the incidence generally, but they have also increased the risk of cardia stomach cancer (Wroblewski et al., 2010[216]). *H. pylori* infection, dietary practices, salt intake, and genetic characteristics are risk factors. More precise medicine may be administered thanks to molecular subtyping developments and genetic testing, which allows for earlier diagnosis (Kusters et al., 2006;[110] Li et al., 2022[123]). The illness still poses a significant threat to public health, calling for updated preventative and early detection measures (Rawla and Barsouk, 2019[171]). In Brazil, *H. pylori *infection, certain food habits, and hereditary factors are risk factors for stomach cancer, which affects both men and women equally and has a greater morbidity and death rate in males over 60 (Fitzmaurice et al., 2019[69]). In Kazakhstan, there has been a declining trend in the incidence and mortality of gastric cancer, with an increase in the frequency of early detection and five-year survival rate (Thapa et al., 2023[196]). The study by Lu et al. emphasized the significance of ferroptosis in gastric cancer. Ferroptosis affects the development, incidence, prognosis, and therapeutic resistance of gastric cancer. The function of ncRNAs in gastric cancer etiology and progression, such as circRNA, miRNA and lncRNA was also emphasized by the study (Lu et al., 2022[137]; Zhao et al., 2019[241]). Two proteins that are important in controlling cell survival and death are Bcl-2 and Beclin1 (Thapa et al., 2023[197]). Apoptosis is inhibited by the anti-apoptotic protein Bcl-2, whereas autophagy, a cellular process that helps preserve cellular health by eliminating dysfunctional components, is regulated by Beclin1 (Bhat et al., 2022[25]; Chen et al., 2019[41]). It is possible for the expression of Beclin1 and Bcl-2 to go out of balance in gastric cancer, which would limit autophagy and contribute to enhanced cell survival. According to research by Shang et al., ferroptosis is enhanced via the *miR-508-3p*/Bcl-2/beclin1/SLC7A11 axis, via which circHIPK3 knockdown reduces the resistance to cisplatin of gastric cancer cells. One approach that shows promise for overcoming resistance to cisplatin is to target ferroptosis. It is suggested that serum exosomal circHIPK3 be used as a non-invasive measure to assess gastric cancer patients' cisplatin resistance (Shang et al., 2023[182]). 

One microRNA that is essential for controlling cellular functions is miR-375. It is important for glucose homeostasis, insulin secretion and pancreatic progression (Chen et al., 2019[40]). miR-375 is a tumor suppressor that is often downregulated in many types of cancer, affecting the growth and spread of cells (Chen et al., 2023[36]; Wei et al., 2021[213]). Its complex regulatory network targets many genes linked to signaling cascades and cell cycle regulation (Bertoli et al., 2013[18]). MiR-375 dysregulation has been linked to a number of malignancies, including diabetes, making it a possible target for treatment (Natalicchio et al., 2023[158]). According to Liu et al.'s study, circRPPH1 is essential for the stemness of gastric cancer cells. It affects ferroptosis and controls the expression of *SLC7A11*, a target of miR-375 in gastric cancer. Through the miR-375/*SLC7A11* regulatory axis, CircRPPH1 also enhances the stemness of gastric cancer cells, making it a viable target for comprehending and influencing the course of gastric cancer (Liu et al., 2023[130]). A circRNA called TMEM87A is found on human chromosome 11. Many tissues, including the heart, brain, and lungs, express circTMEM87A (Chen et al., 2017[44]; Cooper et al., 2020[49]). It has been demonstrated that circTMEM87A interacts with the TMEM87A protein, albeit its exact role is yet unknown (Hirata et al., 2015[94]). A transmembrane protein called TMEM87A is important in controlling cell division and proliferation (Wang et al., 2021[206]). The function of circTMEM87A in the advancement of gastric cancer was examined by Dong et al. The study revealed that GC tissues and cells have decreased miR-1276 and higher levels of circTMEM87A and *SLC7A11*. circTMEM87A functions as a miR-1276 sponge by suppressing cell migration, apoptosis, proliferation, and ferroptosis. The study revealed that circTMEM87A promotes cell migration and proliferation while inhibiting apoptosis (Dong et al., 2024[59]; Mao et al., 2023[146]).

A strong and extremely selective alpha-2 adrenergic agonist having anxiolytic, analgesic, and sedative effects is dexmedetomidine (Giovannitti et al., 2015[81]). When used in critical care and anesthesia settings, it creates a special kind of sedation that encourages compliant and readily arousable patients (Cui et al., 2022[52]; Hughes et al., 2012[96]). Decomedetomidine, in contrast to conventional sedatives, induces conscious sedation that preserves circulatory stability while permitting speech (Kaur and Singh, 2011[106]). Because of its sympatholytic properties, which lower stress reactions, it can be used during a variety of medical operations (Naaz and Ozair, 2014[156]). Research is being done to examine the neuroprotective properties of dexmedetomidine, which shows promise for uses other than sedation (Xu et al., 2022[224]). Despite the favorable profile, cautious observation is required due to the risk of bradycardia and hypotension (Backer et al., 1997[10]; Wang et al., 2021[212]). Gao et al. investigated the effects of dexmedetomidine on gastrointestinal cancer cells and found that it inhibited tumor development *in vivo* and had inhibitory effects on cell survival and apoptosis *in vitro.* In GC cells, dexmedetomidine causes ferroptotic cell death characterized by elevated amounts of iron and reactive oxygen species as well as reduced glutathione. Dexmedetomidine upregulates miR-302a while downregulating circ0008035 and E2F7, indicating that the circ0008035/miR-302a/E2F7 axis might be a target for future therapy (Chen et al., 2021[39]; Gao and Wang, 2023[77]). A transmembrane E3 ubiquitin ligase, zinc and ring finger 3 (*ZNRF3*) controls several important signaling pathways, such as Wnt/β-catenin (Basham et al., 2019[13]). Its main role is to ubiquitinate Frizzled receptors, which are essential for Wnt signaling, and then degrade them (Liu et al., 2022[129]; Yan et al., 2024[225]). *ZNRF3* functions as a negative regulator, regulating Wnt signaling's length and amplitude (Hao et al., 2012[91]). This affects a variety of cellular processes, including tissue homeostasis, differentiation and proliferation (Ruijtenberg and van den Heuvel, 2016[177]). *ZNRF3* dysregulation has been linked to a number of malignancies, where aberrant Wnt signaling plays a role in oncogenesis (Aggarwal et al., 2020[2]; Hao et al., 2016[90]). The function of circ_0000190 in the evolution of gastric cancer was investigated by Jiang et al. In gastric cancer tissues and cell lines, Circ_0000190 expression is reduced, and low expression indicates a bad prognosis. Overexpression encourages ferroptosis while preventing cell invasion, migration, and proliferation. Through its association with *ZNRF3*, Circ_0000190 functions as a sponge for miR-382-5p, inhibiting the advancement of gastric cancer. Overexpression of Circ_0000190 inhibits the development of xenograft tumors *in vivo* (Alharbi et al., 2021[5], Jiang et al., 2022[102]).

### Esophageal cancer

The esophagus, a muscular tube that connects the throat and stomach, is where esophageal cancer begins (Crolla et al., 1991[51]). It usually shows signs such as trouble swallowing and unexpected weight loss at late stages. Squamous cell carcinoma and adenocarcinoma are the two main kinds (Rice et al., 2017[173]). Stomach acid reflux disease (GERD), excessive alcohol use, and tobacco usage are major danger signs. Radiation therapy, chemotherapy, and/or surgery are used as treatments (Bhat et al., 2022[25]; Ness-Jensen and Lagergren, 2017[159]). The stage of the cancer at diagnosis affects the prognosis (Patkunarajah et al., 2020[165]). Regular tests for persons who are at risk are important because they emphasize the roles that early identification and lifestyle adjustments play in prevention (Bhat et al., 2023[26]; Miller, 1981[151]). At fewer than 20 % five-year survival, esophageal cancer is a major worldwide health problem, accounting for about 600,000 new cases diagnosed each year (Domper Arnal et al., 2015[58]). Geographically, esophageal cancer incidence varies; around half of all esophageal cancer cases worldwide are identified in China, where the esophageal squamous cell carcinoma (ESCC) subtype accounts for the bulk of cases (Chellappan et al., 2018[35]; Liang et al., 2017[128]). The overall incidence rate of esophageal cancer is declining globally, which can be ascribed to a number of causes including improving dietary practices, declining rates of drinking and smoking in some areas, and economic prosperity (Gupta et al., 2021[87]; Xu et al., 2020[223]). Diet, alcohol and cigarette usage, esophageal burns, achalasia of the heart, and several viral infections are risk factors for esophageal cancer (Kamangar et al., 2009[105]). According to Xi et al.'s research, circBCAR3 suppression prevents esophageal cancer tumor development and metastasis. QKI functions as a positive regulator when CircBCAR3 interacts with miR-27a-3p to upregulate transportin-1. QKI is transcriptionally activated by hypoxia-induced E2F7, which enhances carcinogenesis and promotes the development of circBCAR3. These results suggested circBCAR3 as a possible target for esophageal cancer treatment (Gupta et al., 2018[88]; Xi et al., 2022[220]). Wnt/β-catenin is an essential signaling cascade that controls several biological functions (Pai et al., 2017[163]). The process of activation starts when the Wnt ligand attaches itself to its receptor, starting a signaling cascade that stops β-catenin from degrading (Gupta et al., 2018[89]; MacDonald et al., 2009[142]). Once stabilized, β-catenin moves into the nucleus and engages in transcription factor interactions to enhance the expression of target genes (Valenta et al., 2012[201]). This route is essential for maintaining stem cells, maintaining tissue homeostasis, and promoting embryonic development (Blanpain and Fuchs, 2009[29]). Dysregulation has been linked to a number of illnesses, including cancer. Gaining knowledge about Wnt/β-catenin signaling can help treat disorders caused by abnormal cellular processes and provide insights into biology during development (Hussain et al., 2024[97]; White et al., 2012[214]). Yao et al. investigated the function of circPVT1 in esophageal cancer 5-FU chemosensitivity. 5-FU-resistant cells have increased CircPVT1 expression, which can be downregulated to improve 5-FU chemosensitivity through decreased multidrug-resistant protein levels and increased cytotoxicity. Additionally, it affects the expression of Frizzled3, which may be overexpressed or inhibited by a miR-30a-5p inhibitor. Because of its involvement in esophageal cancer 5-FU chemosensitivity, CircPVT1 may one day be used as a therapeutic target (Hussain et al., 2023[98]; Yao et al., 2021[229]).

### Lung adenocarcinoma

**Figure 3:**
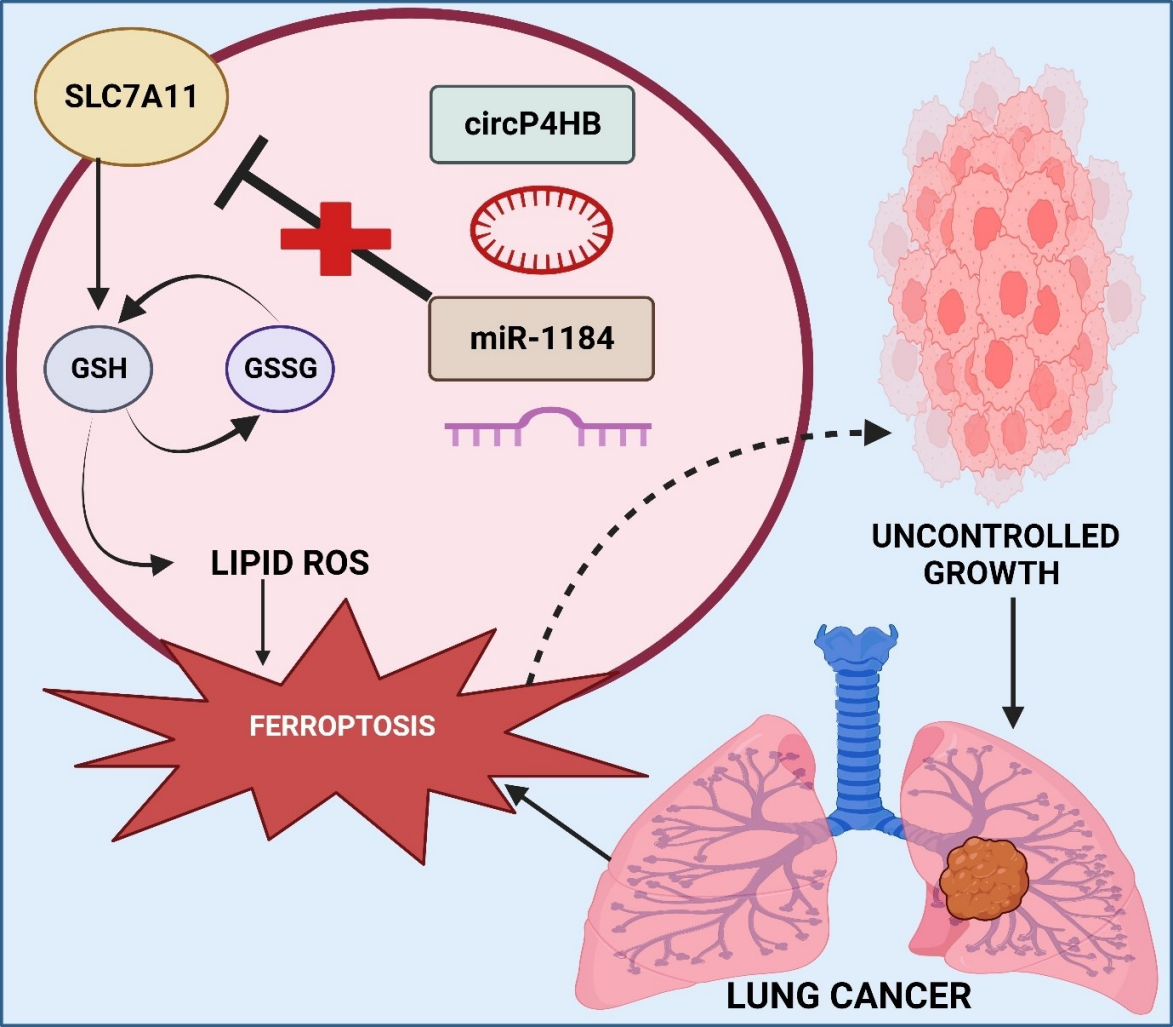
As illustrated in the figure, circular RNA of prolyl 4-hydroxylase, beta polypeptide (CircP4HB) emerges as a potent ferroptosis suppressor in lung adenocarcinoma (LUAD). Elevated levels of CircP4HB in LUAD play a pivotal role in preventing cells from undergoing erastin-induced ferroptosis. This protective mechanism is attributed to the induction of glutathione production by CircP4HB. Furthermore, *in vivo* studies confirm that the overexpression of circP4HB not only enhances tumor development but also effectively suppresses ferroptosis.

One common subtype of NSCLC that starts in the peripheral lung tissues is lung adenocarcinoma (Bhat et al., 2023[27]). It is characterized by acinar or glandular characteristics and often appears on imaging as a peripheral mass (Chen et al., 2022[45]). It usually affects those who have never smoked and those who have smoked in the past. It is frequently linked to mutations like *EGFR* or *KRAS* (Rohilla et al., 2023[174]; Takamochi et al., 2013[192]). Shortness of breath, coughing, and chest discomfort are possible symptoms. Imaging, biopsies, and molecular tests are all part of the diagnosis (Thapa et al., 2023[195]). The range of treatment options includes immunotherapy, targeted treatments, chemotherapy, and surgery (Lemjabbar-Alaoui et al., 2015[117]). The stage at diagnostic determines the prognosis, with early discovery greatly increasing the likelihood of a successful intervention and survival (Denisenko et al., 2018[56]; Rohilla et al., 2023[175]). People who live in highly polluted cities have a greater risk of lung adenocarcinoma; non-smokers are more vulnerable than smokers, according to retrospective research conducted in North China (Dela Cruz et al., 2011[55]). The study also discovered that the cancer was more likely to develop in the right lung and that the majority of individuals with lung adenocarcinoma had no prior history of lung-related conditions (Metwally et al., 2022[150]). Additionally, a different study reported on the rising frequency of non-smoking lung adenocarcinoma as well as the changing clinical features of ground glass opacity (GGO) lung adenocarcinoma patients, suggesting a changing epidemiological profile (Li et al., 2020[127]; Singhvi et al., 2018[186]). These results highlight how crucial it is to comprehend how clinical characteristics and risk factors for lung adenocarcinoma are evolving in order to effectively prevent and treat the disease (Li et al., 2022[119]). Zhang et al. used exosomes and circRNA_101093 (cir93) to investigate ferroptosis resistance in LUAD. Exosomes lower lipid peroxidation, which desensitizes LUAD cells to ferroptosis. Cir93 regulates arachidonic acid by interacting with *FABP3*. In pre-clinical *in vivo* models, ferroptosis-based therapy is improved by inhibiting exosome release (Zhang et al., 2022[239]). The gene SLC7A11, often referred to as xCT, produces a component of the cystine/glutamate transporter. System xCT is the name of this transporter, which is essential to maintaining cellular redox balance (Hutchinson et al., 2019[99]). In the context of ferroptosis, a controlled cell death process marked by iron-dependent lipid peroxidation, *SLC7A11 *is very important (Koppula et al., 2018[107]; Subramaniyan et al., 2022[190]). The SLC7A11-encoded protein aids in the absorption of cystine by cells, which is a necessary precursor to glutathione and vital for shielding cells from oxidative damage (Nguyen et al., 2022[161]). Because it can make cancer cells more susceptible to therapies that cause ferroptosis, inhibition of *SLC7A11 *has been investigated as a therapeutic approach in several malignancies (Lee and Roh, 2022[114]; Thapa et al., 2023[199]). Pan et al. investigated the function of circP4HB in ferroptosis and LUAD. CircP4HB is increased in LUAD and prevents cells from going through erastin-induced ferroptosis by inducing the production of glutathione. The overexpression of circP4HB is confirmed to enhance tumor development and suppress ferroptosis *in vivo*, indicating that it may be a potential biomarker for LUAD (Pan et al., 2022[164]) (Figure 3(Fig. 3)). 

A circRNA known as CircDTL has been connected to the development of NSCLC tumors. According to research by Shanshan et al., CircDTL functions as an oncogene, stimulating the formation of tumors, and is increased in NSCLC cells. It has also been demonstrated to control ferroptosis and apoptosis in NSCLC cells. The study found that GPX4 inhibits both ferroptosis and apoptosis, and that CircDTL causes cancer through the circDTL/miR-1287-5p/GPX4 axis. It has been discovered that squelching CircDTL increases NSCLC cells' susceptibility to chemotherapeutic drugs and prevents tumor development *in vivo*. According to this study, CircDTL may be a useful therapeutic target for the treatment of NSCLC cancer (Shanshan et al., 2021[183]).

### Thyroid cancer

The thyroid gland, an essential component of the endocrine system, is the source of thyroid cancer (Bauer, 2020[14]). It frequently appears as a painless lump in the neck and is characterized by aberrant cell proliferation (Grimm, 2022[84]). Thyroid cancer comes in two primary forms: follicular and papillary, both of which often have good prognoses (Bhat et al., 2024[23]). On the other hand, anaplastic thyroid carcinoma is less treatable and more aggressive. Voice changes and trouble swallowing are common symptoms. Imaging and biopsies are used in diagnosis (Nguyen et al., 2015[160]). Radiation therapy with iodine therapy, surgery, and hormone replacement therapy are available as treatments. The prognosis varies, although results are greatly improved with early diagnosis (Laha et al., 2020[111]). For thyroid cancer to be adequately managed, follow-ups after therapy and routine monitoring are crucial (Carballo and Quiros, 2012[34]). Thyroid cancer's epidemiology has been mostly consistent in recent years, with older persons seeing a rising incidence and its prevalence being greater in women (Maniakas et al., 2022[145]). Numerous risk variables, such as age, gender, and environmental factors including radiation exposure and food, have been discovered (Rahbari et al., 2010[169]). Differentiated thyroid cancer (DTC) and undifferentiated thyroid cancer (UTC) are two of the many subtypes of this complicated illness (Gazeu et al., 2020[78]). While UTC is more aggressive and has a poorer prognosis, DTC is more frequent and has a better prognosis (Paulson et al., 2019[167]). Most thyroid malignancies are detected at an early stage, and improvements in diagnostic and therapeutic methods have raised the overall survival rate for both DTC and UTC (Al-Qurayshi et al., 2021[6]). The function of circ_0067934 in thyroid cancer cells was investigated by Wang et al. Their findings showed that circ_0067934 reduces ferroptosis, which affects indicators like Fe^2+^, iron, and ROS, ultimately decreasing cell viability. By sponging and suppressing miR-545-3p, silencing circ_0067934 causes apoptosis, prevents proliferation, and increases the ferroptosis-negative regulator SLC7A11. The consequences of silencing can be reversed by overexpressing *SLC7A11 *or blocking miR-545-3p, circ_0067934 being proposed as a possible therapeutic target (Wang et al., 2021[207]). Glutathione peroxidase 4, or GPX4, is a vital enzyme that helps shield cells from oxidative damage (Bersuker et al., 2019[17]). It is essential for preserving the integrity of cells since it scavenges and neutralizes dangerous ROS (Li et al., 2021[124]). Lipid peroxidation is a process where free radicals harm cell membranes; GPX4 particularly inhibits this damage (Conrad and Friedmann Angeli, 2015[48]). This enzyme is especially important when considering ferroptosis. Conrad et al., showed that GPX4 is a vital enzyme that helps shield cells from oxidative damage. It is essential for preserving the integrity of cells since it scavenges and neutralizes dangerous ROS. Lipid peroxidation is a process where free radicals harm cell membranes; GPX4 particularly inhibits this damage. This enzyme is especially important when considering ferroptosis, a controlled cell death process that involves lipid peroxidation (Chen et al., 2021[42]).

### Colorectal cancer 

One of the most common and deadly tumors in the world is colorectal cancer, which usually starts in the colon or rectum (Eng et al., 2022[62]). Associated with both hereditary and environmental variables, it frequently develops from benign polyps (Bhat et al., 2023[21]). Changes in bowel habits, blood in the stool, and discomfort in the abdomen are among the symptoms (Alzahrani et al., 2021[7]). For successful treatments, early diagnosis through tests such as colonoscopies is essential (Bhat et al., 2023[22]). Depending on the patient's condition and the stage of the cancer, different treatment options include radiation, chemotherapy, and surgery (Mishra et al., 2013[152]). Promising paths are presented by developments in immunotherapy and precision medicine. Risk can be decreased by making lifestyle changes including eating a balanced diet and getting frequent exercise. Campaigns to raise public awareness emphasize the value of tests for early diagnosis, which improves the likelihood of a good outcome (Gray et al., 2020[83]). Globally, colorectal cancer is a major health problem with differing epidemiological features by location. According to estimates, colorectal cancer will claim 460,000 lives in 2018 and cause 1,388,422 new cases. Men and women have a lifetime risk of 1 in 23 and 1 in 26, respectively (Rugge et al., 2015[176]). The high death rate of colorectal cancer is partly due to the fact that most instances are discovered too late. Age, diet, physical activity, obesity, smoking, alcohol use, and a family history of colorectal cancer are the main risk factors for the disease. In the United States, colorectal cancer ranked third among cancers diagnosed in both men and women in 2017 (Servarayan Murugesan et al., 2018[180]). Since the mid-1980s, there has been a general decline in the annual diagnosis rate of colorectal cancer, mostly as a result of greater screening and modifications to risk factors connected to lifestyle. However, since the mid-1980s, rates have been rising by 1 % to 2 % year among those under 50. A little ncRNA molecule called miR-431 is a member of the miRNA family (Rawla et al., 2019[171][172]). These small molecules bind to target genes' mRNA and stop them from being translated into proteins, which is a critical function they perform in controlling gene expression. MiR-431 exhibits dual functionality as either a tumor suppressor or an oncogene, contingent upon the specific biological context (Macfarlane and Murphy, 2010[143]). It inhibits cell invasion and proliferation in certain malignancies, but it also encourages carcinogenesis in others (Li et al., 2022[122]). The influence of circSTIL on ferroptosis and cell proliferation in CRC was examined in the study by Li et al. In CRC tissues, circSTIL is elevated; silencing it causes ferroptosis and decreases cell growth. According to the study, circSTIL inhibits ferroptosis and increases CRC cell proliferation through the miR-431/*SLC7A11 *signaling pathway, indicating possible targets for CRC therapy (Li et al., 2023[125]). An important axis in controlling cell function and development is formed by the metabolic enzyme SCD and the RNA-binding protein ELAVL1. Cell growth and metabolism are impacted by ELAVL1's promotion of SCD expression. Numerous illnesses, including cancer and neurological conditions, are linked to this interaction (Diaz-Muñoz et al., 2015[57]). Therapeutic intervention for various disorders may include targeting this axis. According to research by Long et al., serum expression of circRNA circRHBDD1 is connected with the advancement of CRC and is markedly elevated in CRC tissues and cells. circRHBDD1 silencing increases ferroptotic cell death and RSL3-induced ferroptosis while inhibiting CRC cell migration and proliferation. According to *in vivo* research, circRHBDD1 knockdown suppresses ferroptosis and CRC carcinogenesis, indicating circRHBDD1 as a possible target for therapy (Long et al., 2024[135]). The miR-326 inhibits the expression of the CCL5 gene, which is important for drawing inflammatory cells. This controls inflammation (Shao et al., 2021[184]). However, downregulated miR-326 allows CCL5 to proliferate, enlisting the help of inflammatory forces and maybe exacerbating conditions like fibrosis and arthritis (Das et al., 2014[53]). The function of circABCB10 in rectal cancer was investigated in the Xian et al. investigation. It was discovered that its overexpression stimulates ferroptosis and apoptosis in cancer cells in conjunction with CCL5 and miR-326 downregulation. The investigation also revealed miR-326 as a circABCB10 target, which attenuates the impact of circABCB10 deletion on these processes to some extent. This showed that circABCB10 would be a useful therapeutic target for the treatment of CRC (Xian et al., 2020[221]).

### Breast cancer

Mammary gland cells are the source of breast cancer, a diverse illness. It is typified by unchecked cell proliferation that results in cancerous growths (Fahad Ullah, 2019[63]). Age, hormonal changes, family history, and genetic abnormalities (BRCA1, BRCA2) are common risk factors (Feng et al., 2018[68]). Breast lumps, nipple discharge, and changes in size or form are among the symptoms. For effective therapy, early diagnosis with mammography and routine screenings are essential. Based on the presence or absence of hormone receptors (estrogen, progesterone, and HER2), breast cancer is categorized into subtypes (Shah et al., 2014[181]). Chemotherapy, surgery, hormone therapy, radiation therapy, and targeted treatments are some of the available treatment options (Menta et al., 2018[149]). Precision medicine's ongoing advancements improve tailored treatment strategies, raising overall survival rates and enhancing the quality of life for those who are impacted. Different epidemiological trends contribute to the high global health burden of breast cancer (Arnold et al., 2022[8]). In low- and middle-income nations, it is the primary cause of cancer-related fatalities and the most frequent cancer in women globally. Globally, there were predicted to be 685,000 fatalities and 2.3 million new cases in 2020 (Bagwe-Parab et al., 2020[11]). The signaling pathway known as the STAT3 axis is essential for a number of cellular activities, including as inflammation, immunology, and cancer (Chen et al., 2023[37]). A transcription factor called STAT3 moves to the nucleus when it is activated by cytokines and growth factors, where it controls the expression of targeted genes (Aggarwal et al., 2009[1]). The STAT3 axis is frequently dysregulated in the setting of cancer, which aids in tumor growth, survival, and immune evasion (Ma et al., 2023[141]). Targeting this route for therapeutic action is a focus of cancer study outcomes, as persistent activation of STAT3 has been linked to a number of different malignancies (Lee et al., 2019[113]). Multiple signaling molecules and feedback loops are involved in the complex interactions within the STAT3 axis, which makes it a dynamic regulatory network in cellular physiology and disease (Hu et al., 2021[95]). The study by Zhang et al. investigated circRHOT1's function in the development of breast cancer. Depletion of CircRHOT1 lowers invasion and migration, triggers apoptosis, and decreases cell proliferation. Additionally, it increases iron, Fe^2+^, and reactive oxygen species, strengthening erastin's inhibitory effect on cell development. As a microRNA-106a-5p sponge, CircRHOT1 suppresses miR-106a-5p and prevents ferroptosis. According to the study, miR-106a-5p and circRHOT1 may be useful therapeutic targets for breast cancer (Zhang et al., 2021[237]). Serine/arginine-rich (SR) proteins include SR-rich splicing factor 1 (SRSF1), often referred to as SF2/ASF (splicing factor 2/alternative splicing factor) is essential for the process of pre-mRNA splicing, which joins exons to create mature mRNA by removing non-coding introns (Bogaert et al., 2023[30]). Particularly, SRSF1 affects alternative splicing, which adds to the variety of mRNA isoforms (Du et al., 2021[61]). A number of illnesses, including cancer, have been linked to aberrant *SRSF1* expression and activity (Lei et al., 2023[116]). *SRSF1* has the ability to influence whether exons are included or excluded in cancer cells, resulting in changed mRNA isoforms that may have carcinogenic qualities (Lv et al., 2021[139]). Using SR-rich splicing factor 1 (*SRSF1*), Song et al. investigated the molecular basis of cisplatin chemosensitivity in TNBC. They discovered that downregulating SRSF1 causes ferroptosis, decreases viability, and increases DDP chemosensitivity. *GCH1* levels are raised by upregulating circSEPT9, which prevents *GCH1* ubiquitination. By preventing ferroptosis, overexpression of circSEPT9 and *GCH1* reduces DDP chemosensitivity. The study proposed *SRSF1* inhibitors as a possible tactic to increase TNBC patients' treatment effectiveness (Song et al., 2024[188]). A subtype of breast cancer known as HER2-positive, or human epidermal growth factor receptor 2-positive, is defined by the overexpression or amplification of the HER2 gene (Iqbal and Iqbal, 2014[100]). One protein involved in cell division and development is called HER2, and overexpression of this protein can result in tumor growth that is unchecked and aggressive. About 20 % of cases of breast cancer are of this subtype (Mo et al., 2022[153]). There are certain therapeutic modalities linked to HER2-positive breast cancer (Jiang et al., 2023[101]). Patients with HER2-positive breast cancer respond considerably better to targeted treatments like pertuzumab and trastuzumab (Herceptin), which work by inhibiting the function of the HER2 protein (Sun et al., 2022[191]). Determining the HER2 status of a breast cancer tumor is crucial in order to optimize therapy options and enhance patient outcomes (Gajria and Chandarlapaty, 2011[74]). Bazhabayi et al., investigated circGFRA1's function in breast cancer. It was discovered that circGFRA1 may be knocked down to prevent cell migration, invasion, and proliferation in HER-2-positive BC. By attaching to miR-1228 and reducing its inhibitory action on AIFM2, CircGFRA1 reduces its effects via a ceRNA mechanism (Bazhabayi et al., 2021[15]).

### Hepatocellular carcinoma 

**Figure 4:**
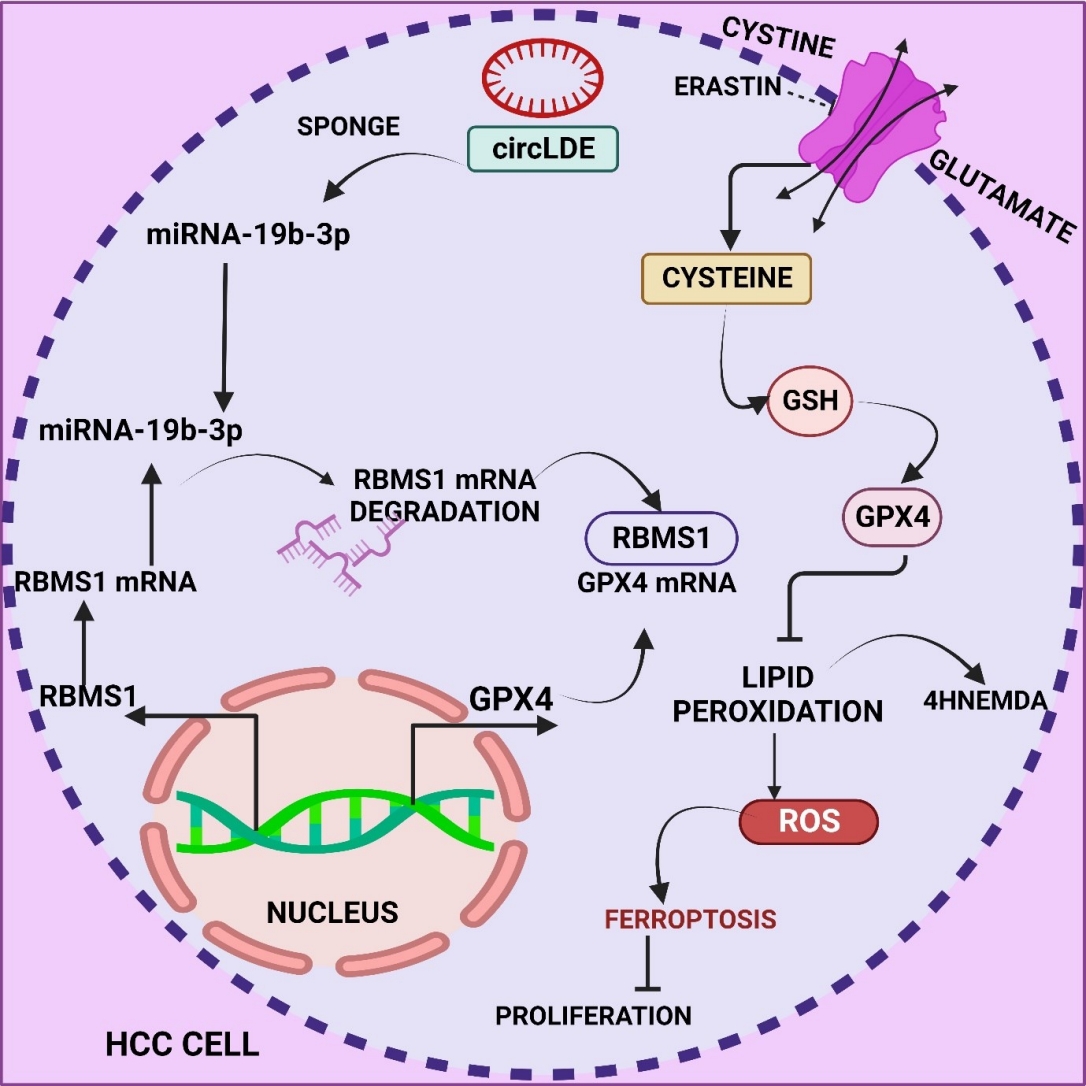
This image depicts the intricate role of RNA binding motif single stranded interacting protein 1 (RBMS1) in hepatocellular carcinoma progression, unveiling its link to ferroptosis regulation. The Circular RNA Intercellular DElay (circIDE)/miR-19b-3p/RBMS1 axis emerges as a potential therapeutic target and prognostic factor in hepatocellular carcinoma.

The most prevalent kind of primary liver cancer is called HCC, and it starts in the major cell type of the liver, called hepatocytes (Alawyia and Constantinou, 2023[4]). It is frequently linked to long-term liver conditions such non-alcoholic fatty liver disease, viral hepatitis (B and C) and cirrhosis. Abdominal discomfort, weight loss, jaundice, and edema are some signs of HCC (Salazar and Le, 2021[179]). Blood tests, imaging investigations, and occasionally a liver biopsy are used in the diagnosis process (Forner et al., 2018[70]). Depending on the cancer's stage, hepatocellular carcinoma patients may get surgery, liver transplantation, ablation treatments, chemoembolization, targeted medicines, immunotherapy, and more (Hennedige and Venkatesh, 2013[93]). The prognosis varies, although better results are possible with early discovery and management. For the purpose of early identification and prompt treatment, people who are at high risk of developing liver cancer must undergo routine monitoring (Guan et al., 2021[86]). A serious worldwide health problem is HCC, which is more common in areas where chronic hepatitis B and C infections are common. It ranks as the fourth most prevalent cause of cancer-related deaths globally and the sixth most common kind of cancer (Yang et al., 2019[226]). Liver cancer claimed the lives of an estimated 841,000 people in 2018; East Asia and sub-Saharan Africa had the highest incidence rates (Ganesan and Kulik, 2023[75]). Alcohol use, aflatoxin exposure, chronic viral hepatitis, and non-alcoholic fatty liver disease are the main risk factors for HCC. For the prevention of HCC, it is essential to identify and treat underlying liver problems as soon as possible (Starzyńska, 2007[189]). According to a study by Lyu et al., circ0097009 is markedly increased in HCC, prevents cell invasion and proliferation. Additionally, it controls the important ferroptosis regulator *SLC7A11* via sponging miR-1261 in HCC. They highlighted the regulatory role of circ0097009 in cancer cell ferroptosis and proposed it as a possible diagnostic biomarker and therapeutic target for HCC (Lyu et al., 2021[140]). A family of medications known as tyrosine kinase inhibitors (TKIs) targets and inhibits certain tyrosine kinases (Thapa et al., 2023[198]). These enzymes are essential for many biological functions, such as signaling, cell division, and growth. Tyrosine kinases that are activated abnormally are frequently linked to specific kinds of cancer (Paul and Mukhopadhyay, 2004[166]). TKIs obstruct the signaling pathways that encourage the development of cancer cells in cancer therapy (Gilles et al., 2022[80]). Their mechanism of action involves obstructing tyrosine kinase activity, which in turn prevents downstream signaling that advances tumor growth (Bhat et al., 2023[20]). TKIs are indicated for the treatment of several cancers, such as chronic myeloid leukemia (CML), breast cancer, gastrointestinal stromal tumors (GISTs), and lung cancer (Shyam Sunder et al., 2023[185]). Bi et al. (2023[28]) discovered that when multi-targeted tyrosine kinase inhibitors, such as Lenvatinib, are administered to hepatocellular carcinoma cells, circular RNA FAM134B (circFAM134B) promotes ferroptosis. CircFAM134B increases the amounts of reactive oxygen species, Fe^2+^, and malondialdehyde in HCC cells via targeting endoplasmic reticulum-phagy. It is a potential therapeutic target because it interacts with PABPC4, an antagonist of nonsense-mediated mRNA decay, to affect the decay of FAM134B mRNA (Bi et al., 2023[28]). The biochemical pathway including GPX4, an enzyme essential for shielding cells from oxidative damage, is known as the GPX4 axis (Chen et al., 2023[38]). One important factor in halting lipid peroxidation is GPX4. The GPX4 axis is especially important when considering ferroptosis, a controlled cell death process marked by lipid peroxidation (Bebber et al., 2020[16]). The metabolism of lipid molecules and the cellular antioxidant system are two of the many elements that make up the GPX4 axis (Wang et al., 2021[210]). In some pathological situations, such as cancer and neurological illnesses, ferroptosis can be triggered by inhibition of GPX4 (Fuloria et al., 2021[73]). Xu et al. investigated ferroptosis, an iron-dependent mechanism of cell death, and its relationship to circIL4R's function in HCC. HCC tissues and cells exhibit a considerable upregulation of CircIL4R, and its reduction expedites ferroptosis while inhibiting tumor development. Via miR-541-3p, it controls glutathione peroxidase 4, its target. MiR-541-3p inhibition lessens the impact of circIL4R knockdown in HCC cells. The functions of circIL4R as a ferroptosis inhibitor and tumor promoter in HCC are confirmed by *in vivo* investigations (Xu et al., 2020[222]). Similarly, Zhai et al. showed that downregulation of RBMS1 in HCC tissues correlates with poorer patient survival. *RBMS1* overexpression inhibits HCC cell growth by suppressing GPX4, promoting ferroptosis via circIDE/miR-19b-3p/*RBMS1* axis in HCC (Zhai et al., 2023[234]) (Figure 4(Fig. 4)).

### Cervical cancer 

A particular kind of cancer called cervical cancer starts in the cells of the cervix, which is the area where the uterus meets the vagina. Human papillomavirus (HPV) infection with high-risk strains is the main cause of cervical cancer (Burd, 2003[32]). Early detection and management are made possible by routine screening with Pap smears or HPV tests (Buskwofie et al., 2020[33]). Abnormal vaginal bleeding, pelvic discomfort, and pain during sexual activity are all possible signs of cervical cancer (Fang et al., 2014[64]). Depending on the cancer's stage, treatment options might include chemotherapy, radiation therapy, and surgery (Vikraman et al., 2022[202]). Cervical cancer incidence has decreased dramatically as a result of preventive measures including HPV vaccination (Moore, 2006[154]). Cervical cancer's epidemiology is notable for having a large worldwide impact, especially in low- and middle-income nations (Brisson et al., 2020[31]). Cervical cancer was expected to have caused 311,000 fatalities and 570,000 new cases in 2018, with sub-Saharan Africa, South-Central Asia, and South-East Asia having the greatest incidence rates (Olusola et al., 2019[162]). Persistent infection with high-risk HPV types is substantially associated with the illness. Disparities in access to therapies such as HPV vaccination and screening, which are effective preventative approaches, add to the uneven Cumber of cervical cancer (Zhang et al., 2020[238]). Reducing the incidence and death of cervical cancer requires ongoing efforts to enhance screening programs and vaccine coverage, especially in areas with low resources (Choi et al., 2022[47]). CircRNA ringmaster circACAP2 shows off its skills in muscle and brain cells. It inhibits the growth of harmful aggregates that can damage neurons by binding to a protein known as FUS (Mehta et al., 2020[148]). The influence of circACAP2 on ferroptosis and its significance in cervical cancer were investigated in the work by Liu et al. By lowering ROS, iron, and Fe^2+^ levels, CircACAP2 reduces the viability of cervical cancer cells. By acting as a rival endogenous RNA to miR-193a-5p, it increases the expression of GPX4. CircaCAP2 knockdown reduces cell viability; this effect can be reversed by overexpressing GPX4 or inhibiting miR-193a-5p (Liu et al., 2022[134]). CircRNA mole-cule circEPSTI1, which is just 100 words long yet has a powerful impact on illness studies, has recently come to light. It functions like to a microscopic investigator, detecting and maybe affecting the EPSTI1 gene's function in cell proliferation (Gu et al., 2023[85]). According to Wu et al.'s study, circEPSTI1 is significantly overexpressed in cervical cancer, which affects cell ferroptosis and lessens the effects of ferritin. The circEPSTI1-miR-375/ 409-3P/515-5p-SLC7A11 axis is linked to ferroptosis and affects the growth of cervical cancer. CircEPSTI1 was proposed by the study as a possible cervical cancer indicator and therapeutic target (Wu et al., 2021[217]).

### Bladder cancer

One kind of cancer that starts in the bladder's cells is called bladder cancer. Transitional cell carcinoma, which originates in the bladder's innermost lining, is the most prevalent kind of bladder cancer (Thapa et al., 2023[199]). Age, frequent bladder infections, smoking, and chemical exposure are risk factors for bladder cancer (Farling, 2017[66]). Frequent urination, pelvic discomfort, and blood in the urine are common symptoms. Urine cytology, imaging investigations, and cystoscopy are just a few of the tests used in the diagnosis process (Thapa et al., 2023[194]). Depending on the illness's stage, bladder cancer patients may get radiation treatment, chemotherapy, immunotherapy, or surgery (Wilson et al., 2022[215]). The prognosis varies, although results are greatly improved by early discovery and treatment. Bladder cancer's epidemiology is typified by its substantial worldwide influence, especially in industrialized nations (Thapa et al., 2022[200]). Globally, bladder cancer was expected to have caused 168,000 deaths and 436,000 new cases in 2018 (Saginala et al., 2020[178]). Numerous risk factors, including as alcohol intake, tobacco use, and occupational exposure to certain chemicals, are highly associated with the condition. There is significant regional variation in the incidence rates, with larger rates found in North America and Europe (Pelucchi et al., 2006[168]). Although mortality rates have decreased in many high-income countries as a result of early identification and treatment, problems still exist in low- and middle-income countries (Wang et al., 2015[203]). The MAPK pathway is an essential cell signaling cascade that governs a number of different biological functions (Fang and Richardson, 2005[65]). The route is started by extracellular cues like stress or growth factors, and it entails the successive activation of kinases including ERK, MEK, and RAF (Morrison, 2012[155]). When activated ERK translocates to the nucleus, it affects gene expression and fosters the division, survival, and multiplication of cells (Fecher et al., 2008[67]). Many disorders, including cancer, are linked to dysregulation of the MAPK pathway, whereby aberrant signaling leads to unchecked cell proliferation (Yip and Papa, 2021[231]). Ferroptosis in bladder cancer cells is regulated by circST6GALNAC6, according to research by Wang et al. It was a tumor suppressor at first, but it now encourages erastin-induced ferroptosis. The small heat shock protein 1 (HSPB1) N-terminus is bound by circST6GALNAC6, which inhibits erastin-induced phosphorylation, which is connected to ferroptosis resistance. Protein kinase C increases HSPB1 phosphorylation, which prevents ferroptosis caused by circST6GALNAC6. According to the study, circST6GALNAC6 may be a target for bladder cancer ferroptosis development (Wang et al., 2022[208]). *OTU deubiquitinase 1*, or *OTUB1*, is a protein-coding gene that produces an enzyme that is a member of the protease family that contains the ovarian tumor (OTU) domain (Bhat et al., 2024[19]). As a deubiquitinating enzyme, *OTUB1* contributes to the control of cellular activities that rely on ubiquitin (Rawat et al., 2023[170]). Controlling the ubiquitin-proteasome system, particularly in relation to ubiquitin chain cleavage and deubiquitination, is one of its well-known functions (Singla et al., 2023[187]). Numerous physiological processes, such as immunological response, cellular signaling, and DNA repair, have been linked to *OTUB1* (Fu et al., 2023[72]). According to Wang et al.'s research, trastuzumab resistance in HER2-positive breast cancer is significantly influenced by circ-BGN. Although it may be eliminated to increase cell viability and restore trastuzumab sensitivity, it is correlated with poor overall survival. By interacting with *OTUB1* and *SLC7A11*, Circ-BGN promotes deubiquitination and prevents ferroptosis. This indicated circ-BGN as a new trastuzumab resistance regulator (Wang et al., 2022[211]) (Table 1(Tab. 1)). 

## Conclusion and Future Perspective

Ferroptosis and circRNAs have a major role in cancer treatment. One key technique for cancer therapy and medication resistance has been found to be ferroptosis. By demonstrating its promise in treating several cancer types, such as lung, esophageal, hepatocellular, and breast cancer, targeting ferroptosis is thought to be a developing therapy strategy. CircRNAs have also been shown to have involvement in the development of cancer, they have been identified to promote proliferation and metastasis. Reprogramming cell metabolism in breast cancer has also been linked to exosomal circRNAs. Ferroptosis and circRNAs, in summary, show promise as possible targets for cancer treatment.

However, more research is required to fully understand the terrain of circRNA-mediated ferroptosis regulation in various cancer types. Comprehensive mechanistic investigations are necessary to clarify certain circRNA-mRNA interactions and their influence on ferroptotic pathways. For the purpose of finding viable treatment targets, novel approaches for the profiling and characterization of circRNAs implicated in ferroptosis are needed. A thorough grasp of the complex regulatory networks may be obtained by combining omics data with computational analysis. Additionally, the creation of delivery mechanisms and thorough preclinical validations are necessary to fully explore the translational potential of circRNA-based treatments. The significance of interdisciplinary research teams is highlighted by the necessity of cross-disciplinary collaboration in order to effectively utilize this knowledge for therapeutic applications.

## Conflict of interest

The authors declare no conflict of interest.

## Figures and Tables

**Table 1:**
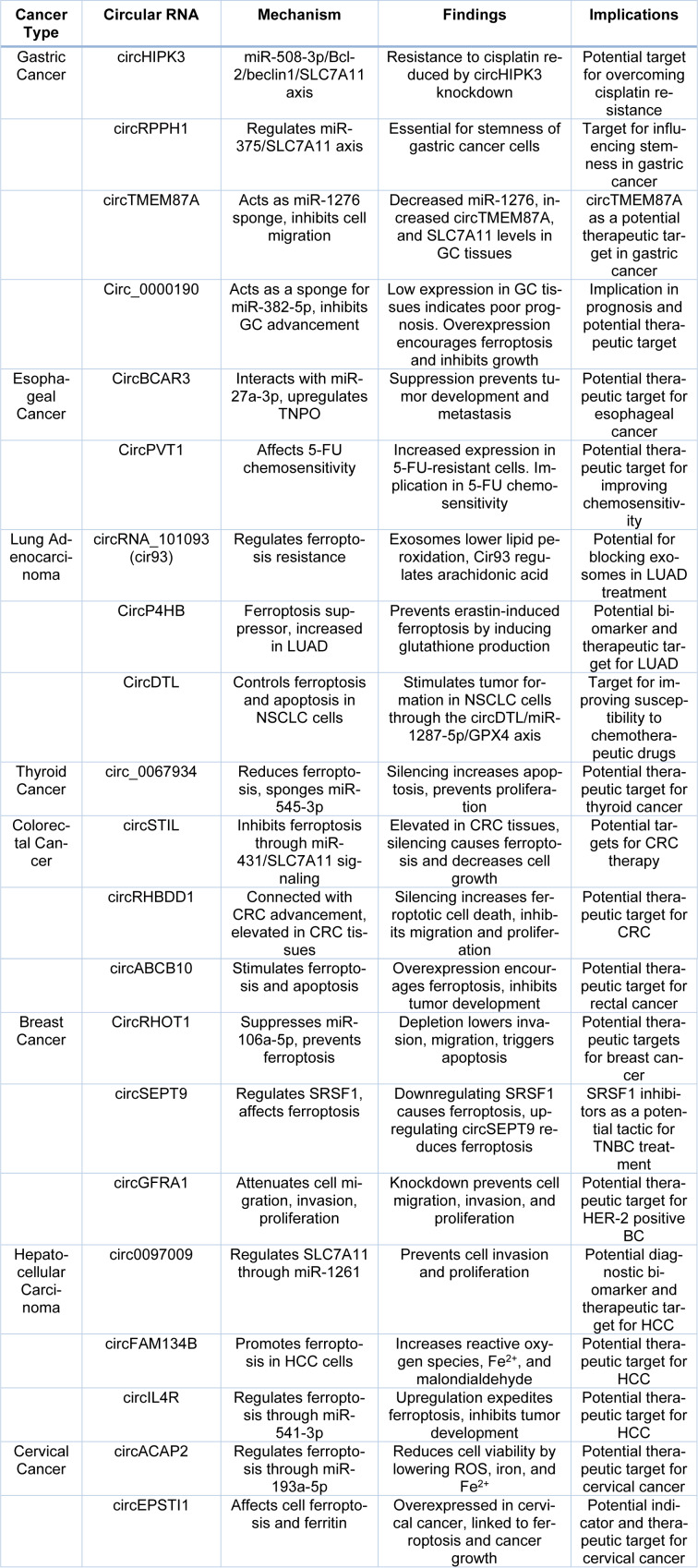
This table summarizes various circular RNAs associated with different cancer types, elucidating their distinct mechanisms, findings, and potential implications. Each study highlights crucial insights, offering a concise overview of the intricate interplay between circular RNAs and cancer-related processes.

**Figure 1:**
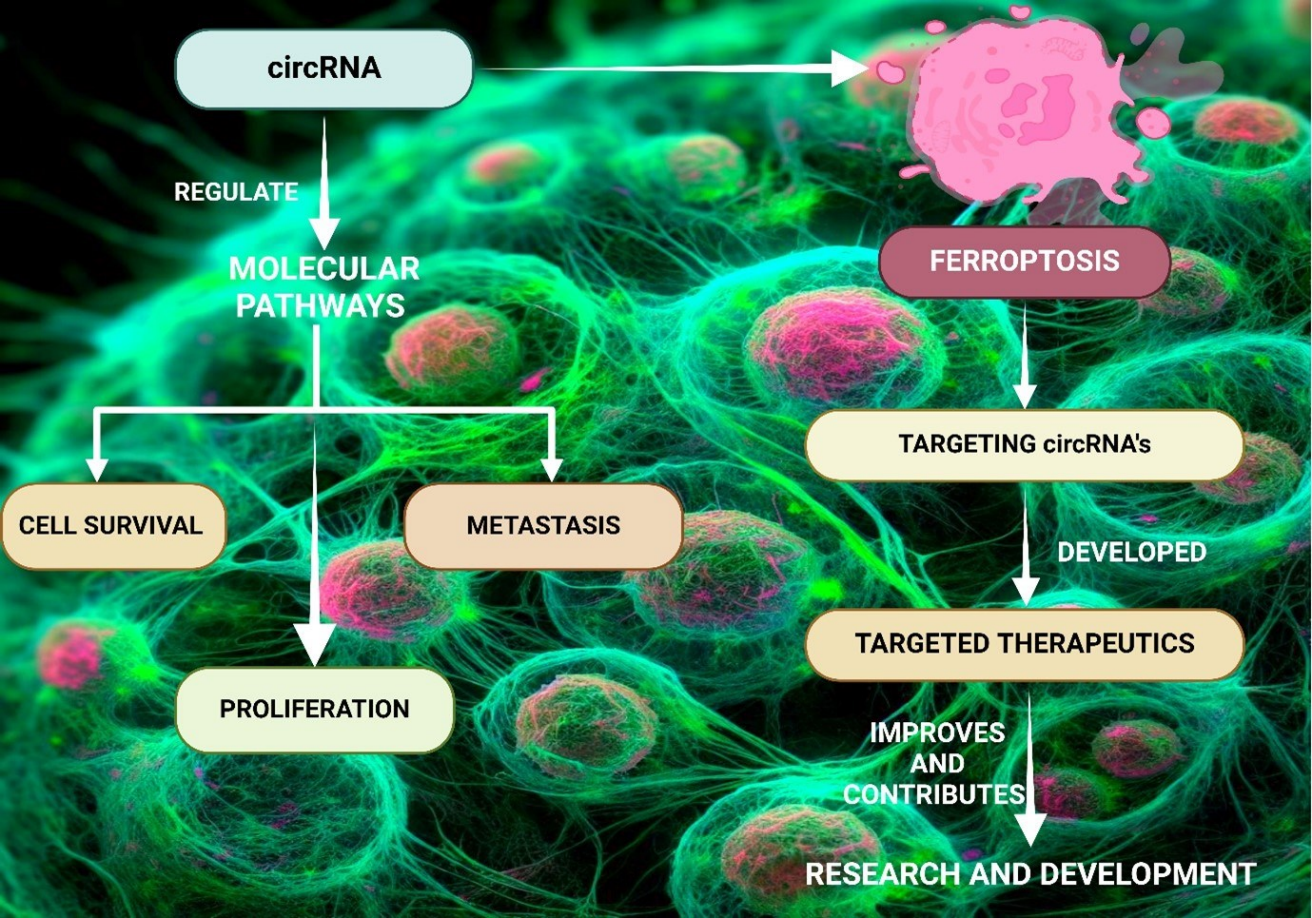
Graphical abstract
